# Annotation of susceptibility SNPs associated with atrial fibrillation

**DOI:** 10.18632/aging.103615

**Published:** 2020-09-09

**Authors:** Chengqi Xu, Rongfeng Zhang, Yunlong Xia, Liang Xiong, Wei Yang, Pengyun Wang

**Affiliations:** 1 College of Life Science and Technology, Center for Human Genome Research and Cardio-X Institute, Huazhong University of Science and Technology, Wuhan 430074, P. R. China; 2 Department of Cardiology, First Affiliated Hospital of Dalian Medical University, Dalian 116011, P. R. China; 3 Department of Clinical Laboratory, Liyuan Hospital of Tongji Medical College, Huazhong University of Science and Technology, Wuhan 430077, P. R. China; 4 Jilin Provincial Key Laboratory on Molecular and Chemical Genetic, The Second Hospital of Jilin University, Changchun 130041, P. R. China

**Keywords:** atrial fibrillation, SNP, annotation, genetics, non-coding

## Abstract

Objective: Genome-wide association studies (GWAS) and the candidate gene based association studies have identified a panel of variants associated with atrial fibrillation (AF), however, most of the identified single nucleotide polymorphisms (SNPs) were found located within intergenic or intronic genomic regions, and whether the positive SNPs have a real biological function is unknown, and the real disease causing gene need to be studied.

Results: The current results of the genetic studies including common variants identified by GWAS (338 index SNPs) and candidate gene based association studies (40 SNPs) were summarized.

Conclusion: Our study suggests the relationship between genetic variants and possible targeted genes, and provides insight into potential genetic pathways underlying AF incidence and development. The results may provide an encyclopedia of AF susceptibility SNPs and shed light on the functional mechanisms of AF variants identified through genetic studies.

Methods: We summarized AF susceptibility SNPs identified by GWAS and candidate gene based association studies, and give a comprehensive functional annotation of all these AF susceptibility loci. by genomic annotation, microRNA binding prediction, promoter activity analysis, enhancer activity analysis, transcription factors binding activity prediction, expression quantitative trait loci (eQTL) analysis, long-range transcriptional regulatory function analysis, gene ontology and pathway enrichment analysis.

## INTRODUCTION

Atrial fibrillation (AF), which is characterized by rapid and irregular beating of the atria, and known as the most common type of cardiac arrhythmia. According to the epidemiologic data, the prevalence of AF ranged from 0.7% to 1% in the general population, and up to 8% in elders greater than 80 years [1, 2]. Meanwhile, AF increases the risk of stroke, congestive heart failure, sudden cardiac death, and increase the rate of substantial morbidity and mortality for about 2 folds [3].

AF is often associated with complications such as hypertension, valvular heart disease, coronary artery disease, heart failure, hyperthyroidism, structural heart diseases, and is also clearly heritable [4, 5]. The important role of genetic factors in the pathogenesis of AF has shown by the identification of AF-causing mutations or rare variants in some families with lone AF, which occurs in structurally normal hearts and without known risk factors [6–8]. Meanwhile, in general AF, the non-hypothesis-driven genome-wide association studies (GWAS) and the candidate gene based association studies have identified a panel of common variants confer risk to AF [9–11]. These studies have set up a key role for the genetic background in generating for AF.

GWAS investigate associations between genomic variants and a disease or trait at the whole genome level without priori assumptions of genomic locations or potential functions of candidate genes. In this case, most of the identified single nucleotide polymorphisms (SNPs) associated with disease were found located within intergenic or intronic genomic regions, and whether the positive SNPs have a real biological function is unknown, and the real target gene need to be further studied [12, 13]. For example, SNP rs2200733 on chromosome 4p25 is the first risk variant for AF identified by GWAS and is the most robustly replicated AF locus to date. The gene that closest proximity to rs2200733 and other AF susceptibility variants in 4q25 is the *PITX2.* Studies in mice showed that *pitx2* haplo-insufficiency promotes an atrial arrhythmia [14]. However, functional evidence about the mechanisms linking these non-coding variants with *PITX2* or the incidence of AF is limited, until a recent study found that these non-coding variants in 4q25 possessing a long-range enhancer–promoter interactions and exert as a transcriptional regulatory directed function at *PITX2* [15]. Understanding the biological nature of non-coding variants associated with AF can enable us to point the real causal genes causing AF and provide insight into the mechanism of AF.

Considering one of the most important challenges of AF genetic study is to elucidate functional mechanisms that how the susceptibility loci modulate AF risk, in the current study, we summarized the results of the studies including variants identified by GWAS and candidate gene based association studies, and give a comprehensive functional annotation of all these AF susceptibility loci. The non-synonymous SNPs were first identified and classified as functional SNPs, and for SNPs in the non-coding region, we try to predict their potential functions including microRNA binding, promoter activity, enhancer activity, transcription factors binding activity, expression quantitative trait loci (eQTL), and long-range transcriptional regulatory function. Our results may provide an encyclopedia of AF susceptibility SNPs and shed light on the functional mechanisms of AF variants identified through genetic studies.

## RESULTS

### AF susceptibility loci

**Figure 1:**
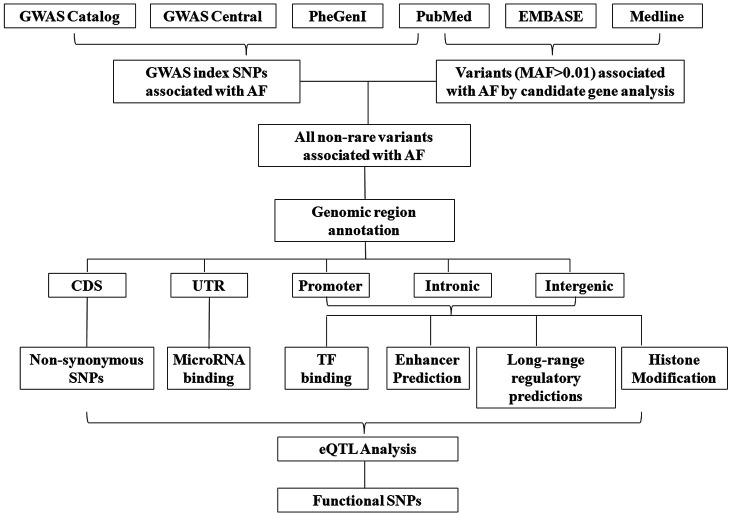
**Workflow of the annotation of susceptibility SNPs associated with atrial fibrillation.**

**Figure 2:**
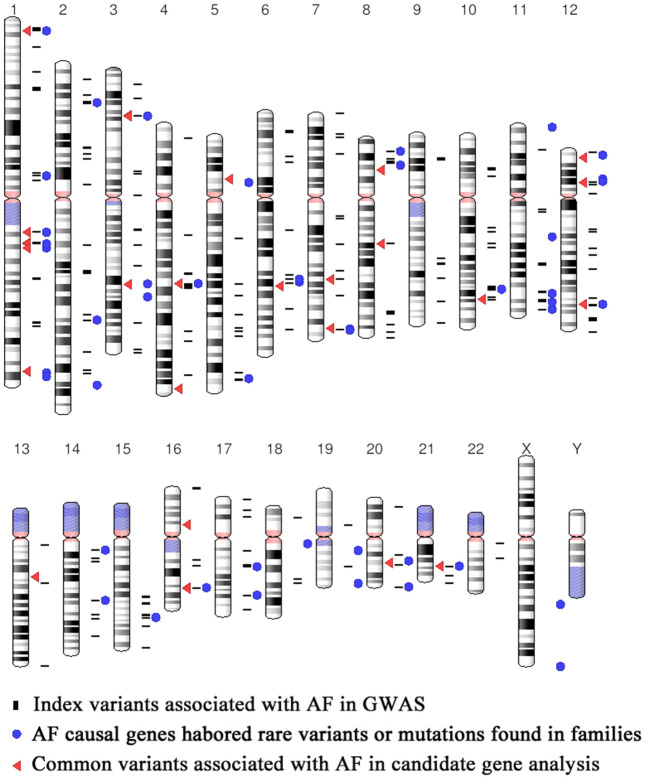
**Distribution of the 378AF susceptibility SNPs and AF causal genes.**

Through searching the public databases including GWAS catalog (https://www.ebi.ac.uk/gwas/), GWAS central (https://www.gwascentral.org), and literatures in Pubmed, Embase and Medline, we included 18 AF GWAS and exome-wide association study (EWAS) in our study, which published from 2007 to 2019 (Table 1). The workflow of the current study is shown in Figure 1. Participants of these studies were mainly European ancestry (15 of 18 studies), and the rest were East Asian (Korean ancestry and Japanese) (Table 1). A total of 338 SNPs (refer as index SNPs) passed the multiple corrections (P<5×10^-8^ or corresponding multiple correction threshold) and showed a significant association with AF in GWAS and EWAS (Figure 2 and Supplementary Table 1). We also include 40 common SNPs which showed significant associated with AF in case control or population prospective study in candidate gene based analysis, or replication study of GWAS loci (Figure 2 and Supplementary Table 1). Totally, we included 378 AF susceptibility SNPs in our further functional annotation.

**Table 1:** Included studies with SNPs associated with AF from 2007 to 2019.

**Number**	**Studies**	**Year**	**Discovery population**	**Replication population**
**enome-wide association study**				
1	Nielsen JB. et al [16]	2018	60,620 European ancestry cases and 970,216 European ancestry controls	NA
2	Roselli C. et al [17]	2018	55,114 European ancestry cases and 482,295 European ancestry controls, 8,180 Japanese ancestry cases and 28,612 Japanese ancestry controls, 1,307 African American ancestry cases and 7,660 African American ancestry controls, 845 Hispanic cases and 4,177 Hispanic controls	NA
3	Nielsen JB. et al [18]	2018	6,337 European ancestry cases and 61,607 European ancestry controls	30,679 European ancestry cases and 278,895 European ancestry controls
4	Thorolfsdottir RB. et al [19]	2018	14,710 cases and 373,897 controls from Iceland, 14,792 cases and 393,863 controls from the UK Biobank	9,204 cases and 76,161 controls, European ancestry
5	Lee JY. et al [20]	2017	672 Korean ancestry cases and 3,700 Korean ancestry controls	200 Korean ancestry cases and 1,812 Korean ancestry controls
6	Christophersen IE. et al [21]	2017	GWAS: 18,398 individuals with atrial fibrillation and 91,536 referents, EWAS: 22,806 AF cases and 132,612 referents.	NA
7	Low SK. et al [22]	2017	8,180 Japanese ancestry cases and 28,612 Japanese ancestry controls	3,120 Japanese ancestry cases and 125,064 Japanese ancestry controls, 15,993 European ancestry cases and113,719 European ancestry controls
8	Thorolfsdottir RB. et al [23]	2017	14,255 AF cases and 374,939 controls, Iceland	2,002 non-Icelandic cases and 12,324 controls
9	Yamada Y. et al [24]	2017	884 patients with atrial fibrillation and 12,282 controls, Japanese	NA
10	Lubitz SA. et al [25]	2016	1,734 individuals with and 9,423 without AF, European ancestry	NA
11	Kertai MD. et al [26]	2015	620 European ancestry cases, 257 European ancestry controls	220 cases and 84 controls
12	Sinner MF. et al [27]	2014	6,707 AF cases and 52,426 controls in Europeans, 843 AF and 3,350 controls in Japanese	6,691 AF cases and 17,144 controls in Europeans, 1,618 AF cases and 17,190 controls
13	Ellinor PT. et al [28]	2012	6,707 European ancestry cases and 52,426 European ancestry controls	5,381 European ancestry cases and 10,030 European ancestry controls
14	Ellinor PT. et al [29]	2010	1,335 European ancestry lone AF cases and 12,844 European ancestry controls	1,164 European ancestry AF cases, 3,607 European ancestry controls
15	Gudbjartsson DF. et al [30]	2009	2,385 European ancestry cases and 33,752 European ancestry controls	2,427 European ancestry cases and 3,379 European ancestry controls, 286 Han Chinese ancestry cases and 2,763 Han Chinese ancestry controls
16	Benjamin EJ. et al [31]	2009	3,413 cases and 37,105 referents, European ancestry	2,145 cases and 4,073 controls, European ancestry
17	Larson MG. et al [32]	2007	151 cases and 1,190 controls from 310 families	NA
18	Gudbjartsson DF. et al [33]	2007	550 European ancestry cases and 4,476 European ancestry controls	3,030 European ancestry cases and 14,780 European ancestry controls, 333 Han Chinese ancestry cases and 2,836 Han Chinese ancestry controls
**Candidate gene based association study**				
19	Cao H. et al [34]	2019	828 patients and 834 controls in Chinese population	NA
20	Xiong H. et al [35]	2019	944 AF patients and 981 non-AF controls in Chinese population	732 cases and 1,291 controls in Chinese population
21	Wang P. et al [36]	2018	1,164 AF patients and 1,460 controls	NA
21	Zaw KTT. et al [37]	2017	452 cases and 1,981 controls in Japanese	NA
22	Feng W. et al [38]	2017	300 AF cases and 300 controls	NA
23	Nakano Y. et al [39]	2016	577 cases and 1935 controls in Japanese	NA
24	Seppälä I. et al [40]	2016	1,834 individuals with AF and 7,159 unaffected individuals	NA
25	Fang Z. et al [41]	2016	1,150 AF cases and 1,150 AF-free controls in Chinese	NA
26	Wang C. et al [42]	2016	1,127 unrelated AF patients and 1,583 non-AF subjects	NA
27	Fang Z. et al [41]	2016	597 AF cases and 996 AF-free controls in Chinese	NA
28	Zhang R. et al [43]	2016	1,132AF patients and 1,206 controls	NA
29	Roberts JD. et al [44]	2016	2,601 incident of AF in a total of 17,529 participant	NA
30	Luo Z. et al [45]	2016	889 AF patients and 1015 controls, Chinese	NA
31	Chen S. et al [46]	2015	941 cases and 562 controls, Chinese	2,113 cases and 3,381 controls
32	Liu Y. et al [47]	2015	597 AF patients and 996 non-AF controls in Chinese	NA
33	Rosenberg MA. et al [48]	2014	879 incident AF in a total 3,309 participants	NA
34	Andreasen L. et al [49]	2014	657 patients diagnosed with AF and a control group comprising 741 individuals	NA
35	Luo Z. et al [50]	2014	889 AF patients and 1,015 controls in Chinese	NA
36	Voudris KV. et al [51]	2014	509 patients of whom 203 with AF	NA
37	Liu Y. et al [52]	2014	597 AF patients and 996 non-AF controls in Chinese	NA
37	Andreasen L. et al [49]	2014	657 AF cases and 741 controls, European ancestry	NA
38	Lin H. et al [53]	2014	948 cases and 3,330 controls, European ancestry	NA
39	Adamsson S. et al [54]	2014	520 incident AF in a total 3,900 subjects, European ancestry	2,247 cases, 2,208 controls
40	Cao H. et al [55]	2014	840 AF patients and 845 controls in Chinese	NA
41	Marott SC. et al [56]	2013	358 patients with lone AF, 299 non-lone AF, and 741 controls, European ancestry	NA
42	Jeff JM. et al [57]	2014	1,288 patients with cardiac surgery, European ancestry	NA
43	VoudrisKv. et al [51]	2014	509 patients with cardiac surgery, European ancestry	NA
44	Ilkhanoff L. et al [58]	2014	241 cases and 3,144 controls, African Americans	NA
45	Marott SC. et al [56]	2013	2,570 AF events in 69,611 participants, European ancestry	NA
46	Andreasen L. et al [59]	2013	358 patients with lone AF and a control of 751 individuals, European ancestry	NA
47	Olesen MS. et al [60]	2012	209 patients with early-onset lone AF, and a control group consisting of 534 individuals free of AF	NA
48	Schnabel RB. et al [61]	2011	European (n=18,524; 2260 AF cases in a total 18,524individuals cohort in European ancestry), 263 AF cases in a total of 3,662 African American descent.	468 AF cases and 438 controls
49	Wirka RC. et al [62]	2011	384 early onset lone AF cases and 3,010 population control	NA
50	Li C. et al [63]	2011	650 AF patients and 1,447 non-AF controls	NA
51	Lubitz SA. et al [64]	2010	790 case and 1,177 control subjects, European ancestry	5,066 case and 30,661 referent subjects, European ancestry
52	Roberts JD. et al [65]	2010	620 AF cases and 2,446 healthy controls	NA
53	Ren X. et al [66]	2010	384 sporadic AF patients and 844 controls	NA
54	Shi L. et al [67]	2009	383 AF patients versus 851 non-AF controls	NA
55	Kääb S. et al [68]	2009	3,508 AF cases and 12,173 controls, European ancestry	NA
56	Sinner MF. et al [69]	2008	1,207 AF-cases and 2,475 controls	NA
57	Giusti B. et al [70]	2007	456 AF patients and 912 matched controls	NA

**Figure 3:**
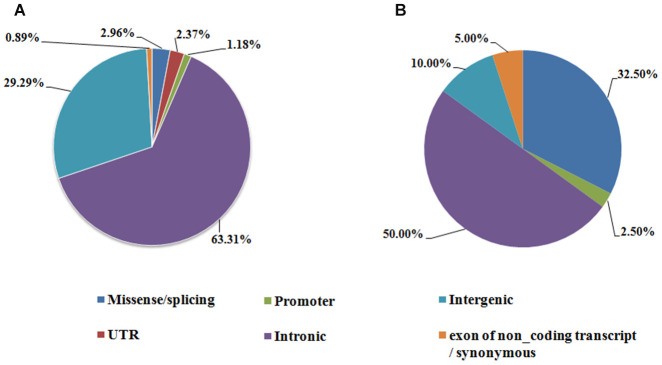
**Genomic region annotation distribution of the AF susceptibility SNPs.** (**A**) Index SNPs associated with AF identified in GWAS, (**B**) AF susceptibility SNPs identified by candidate gene based analysis.

Genomic region annotation using Variant Effect Predictor (http://asia.ensembl.org/Homo_sapiens/Tools/VEP, GRCh38) showed that only a small portion of GWAS index AF SNPs was located in exon of known genes (21/338, 6.21%), 63.31% were found in introns (214/338), and 29.29% locate in intergenic regions (99/338) (Figure 3). In candidate gene based analysis, 50% identified AF related variants locate in intron, and the proportion of non-synonymous variants associated with AF (32.50%, 13/40) was higher than in GWAS index SNPs (2.936%, 10/338) (Figure 3).

### Functional annotation of missense and splicing related SNPs

Through GWAS, EWAS and candidate gene based association study, a total of 36 SNPs were found located in exon of known genes. Missense variants in *SPATC1L* (rs113710653) [24], *TNFSF13* (rs11552708) [24], *SLC22A25* (rs11231397) [24], *RPL3L* (rs140185678) [16], *GCOM1* and *MYZAP* (rs147301839) [16], *UBE4B* (rs187585530) [17], *NEBL* (rs2296610) [22], *LRIG1* (rs2306272) [17], *PLEC* (rs373243633) [23], *DNAH10OS* (rs12298484) [17] were associated with risk of AF through GWAS and EWAS approach, and candidate gene based association study found missense variants in *AGTR1* [38], *AGXT2* [40], *ZFHX3* [52], *MTR* [70], *KCNH2* [69], *KCNE1* [51], *NPPA* [66] confer risk to AF. Two index SNPs of GWAS, including rs140192228 in *RPL3L* [16] and rs133902 in *MYO18B* [16] were predicted may change the mRNA splicing.

### Functional annotation of AF susceptibility SNPs in UTR

8 AF susceptibility SNPs were found in the UTR of protein-coding genes, and 4 of them were predicted to alter the microRNA binding ability predicted by MirSNP (http://bioinfo.bjmu.edu.cn/mirsnp/search) and miRNASNP (http://bioinfo.life.hust.edu.cn/). Rs1049334 in the 3’UTR of *CAV1* was predicted change the binding with hsa-miR-125a-3p, hsa-miR-3620, hsa-miR-4299, hsa-miR-4726-3p, hsa-miR-4783-3p and hsa-miR-497-3p. Rs13385 in the 3’UTR of *HBEGF* was expected to alter the binding with hsa-miR-1207-5p and hsa-miR-4763-3p. Rs7508 in 3’UTR of *ASAH1* may change the binding of hsa-miR-134, hsa-miR-3118, hsa-miR-5190 and hsa-miR-628-5p. Rs951366 in *NUCKS1* was found in the binding region of hsa-miR-3929, hsa-miR-4419b, hsa-miR-4478, hsa-miR-4649-3p and hsa-miR-485-5p.

### Functional annotation of AF susceptibility SNPs in non-coding regions

**Figure 4:**
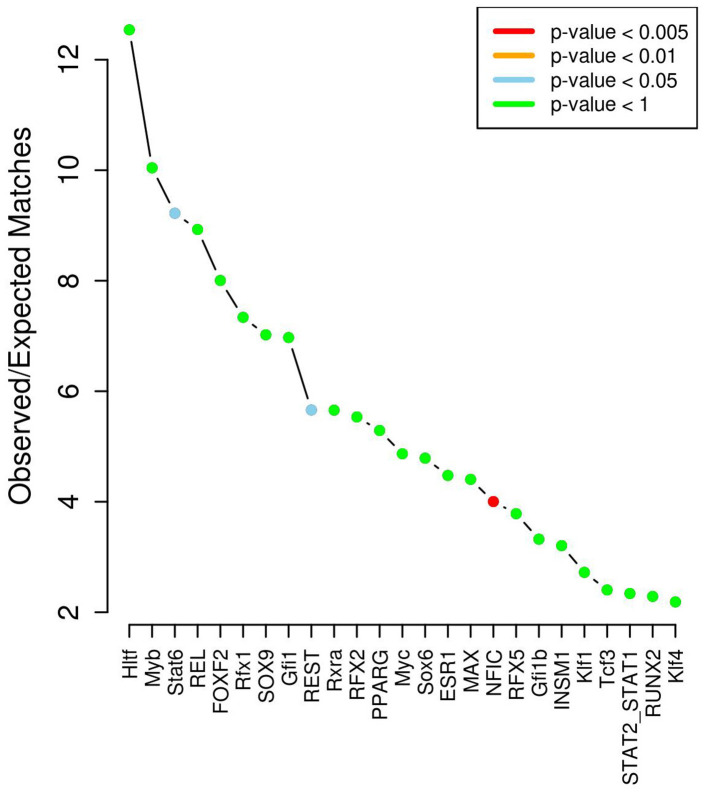
**Transcription factor enrichment analysis results.**

According to the data of the chromatin state and modification of histone binding, a total of 250 SNPs in non-coding regions were identified as located in enhancer regions or might affect the histone mark of promoters and enhancers (Supplementary Table 1), and further analysis found that 65 of them may change the situation of interaction with transcription factors (Supplementary Table 1). 40 transcription factors were found interact with these SNPs. After corrected by genome-wide expected binding ability, these SNPs were significantly enriched for disruption of 3 TFs including STAT6 (*P*=0.02), REST (*P*=0.05) and NFIC (*P*=3.86x10^-3^) (Figure 4).

### eQTL analyses

SNPs in the non-coding region may associate with the expression levels and act as eQTL. We assessed the data from GTEx database (https://gtexportal.org/home/) and evaluate whether AF susceptibility SNPs affect the target gene expression levels. The results showed that 151 SNPs can affect the expression levels of a total of 328 target genes, and 81 of them associated with the expression levels of the closest gene (Supplementary Table 1). Combined with the TF binding data, 39 eQTL effect SNPs were found may alter the binding with transcription factors (Supplementary Table 1).

### Long-range transcriptional regulatory function predictions

We used 3dSNP database (http://cbportal.org/3dsnp/) to analyze whether AF susceptibility SNPs affect distal target genes through topological interactions and function as long-range transcriptional regulatory elements. Results indicated that a total of 211 SNPs interact with distal target genes, and 104 of them exert as an eQTL effect (Supplementary Table 1).

### Gene ontology and pathway enrichment analyses of eQTL targeted genes

**Figure 5:**
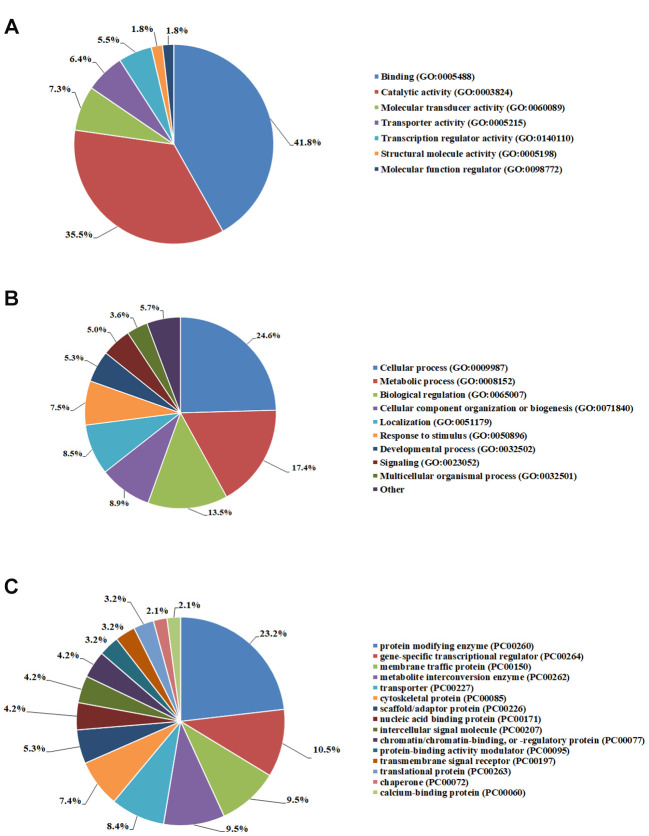
**Gene ontology analyses of AF eQTL targeted genes.** (**A**) Molecular function. (**B**) Biological process. (**C**) Protein class.

eQTL targeted genes of AF were mapped onto Gene ontology (GO) database using three primary categories including molecular function, protein class and biological process via PANTHER (http://www.pantherdb.org). The results showed that AF related genes were mainly enriched in binding, cellular process, metabolic process, protein modifying enzyme, gene-specific transcriptional regulator and membrane traffic protein (Figure 5).

All eQTL targeted genes of AF were subjected to pathway enrichment analysis in the Search Tool for the Retrieval of Interacting Genes (STRING, v11.0, http://string-db.org). Statistical enrichment tests were executed on gene lists within the STRING by Gene Ontology and pathway annotations. The results uncovered some signaling pathway may play roles in AF including “organelle organization”, “striated muscle cell development”, “nuclear migration”, “endomembrane system organization” and “striated muscle cell differentiation” (Table 2).

**Table 2:** Significantly enriched pathways of AF eQTL targeted genes.

**#term ID**	**term description**	**observed gene count**	**false discovery rate**	**matching proteins in your network (labels)**
GO:0006996	organelle organization	56	2.0x10^-4^	*BAZ2A,BRD8,CASQ2,CAV1,CAV2,CDC23,CEP68,CFL2,CHRAC1,CISD2,CTC1,DEK,DNM1L,GATAD1,GORAB,HDAC5,HEATR2,HIP1R,HPS6,IMMT,KANSL1,KDM1B,KIF3C,MAPT,MTHFR,MTSS1,MYH10,MYOZ1,NDUFB10,NEK6,NEURL1,NKX2-5,NR3C1,NSF,NUCKS1,PCID2,PCM1,PEX26,PFDN1,PTK2,RAB29,RAB3IP,REEP1,REEP2, REEP4, RPL3L, SCMH1, SEC24C, SYNE2, TMEM70, UBE2D3, USP36, VPS37B, WIPF1, ZNF462, ZPBP2*
GO:0055002	striated muscle cell development	8	0.03	*CASQ2, CAV2, CFL2, CHRNB1, FLNC, MYH10, MYOZ1, NKX2-5*
GO:0007097	nuclear migration	4	0.04	*MYH10, PCM1, PTK2, SYNE2*
GO:0010256	endomembrane system organization	12	0.04	*CAV1, CAV2, MTSS1, MYH10, NEK6, RAB29, REEP1, REEP2, REEP4, SYNE2, VPS37B, ZPBP2*
GO:0051146	striated muscle cell differentiation	9	0.04	*BMP4, CASQ2, CAV2, CFL2, CHRNB1, FLNC, MYH10, MYOZ1, NKX2-5*

## DISCUSSION

Population-based genetic analysis including GWAS and candidate gene based analysis has identified several SNPs associated with the risk of atrial fibrillation, here, we summarized the current results of the common variants conferred risk to AF and totally including 378 SNPs. Considering most of the AF susceptibility SNPs were located in the non-coding genomic regions, we give a comprehensive functional annotation of all these AF susceptibility SNPs through microRNA binding prediction, promoter and enhancer activity prediction, transcription factors binding activity prediction, eQTL analysis, and long-range transcriptional regulatory function predictions.

Our functional annotation found that 151 AF susceptibility SNPs showed an eQTL effect, and 238 SNPs in non-coding regions were identified as located in enhancer regions or might affect the histone mark of promoters and enhancers, Previous studies also showed that 50-60% of the traits associated non-coding variants identified by GWAS were found located in DNase I hypersensitivity regions [71, 72], and these results also suggest that most of the SNPs identified by the GWAS as predisposing to atrial fibrillation may have biological functions and exert regulatory effects. Our results also showed that only 81 of the 151 eQTL SNPs associated with the expression levels of the closest genes, and a total of 328 target genes were identified affected by AF susceptibility SNPs. Our results identify novel genes that may be associated with the occurrence or development of AF. For example, rs35006907 located in 139bp upstream of a non-coding RNA gene LINC00964, was found associated with the expression level of *MTSS1* gene (P=2.02×10^-18^) in the left ventricle, which 119 kb downstream of rs35006907. Rs35006907 was predicted within an enhancer in several types of tissues including the right ventricle and right atrium, and long-range transcriptional regulatory function predictions also showed that rs35006907 and its located enhancer can interact with *MTSS1* through long-range 3D chromatin loops. *MTSS1* can promote actin assembly at intercellular junctions and a recent functional study indicated that rs35006907 showed a cardioprotective effect [73].

**Figure 6:**
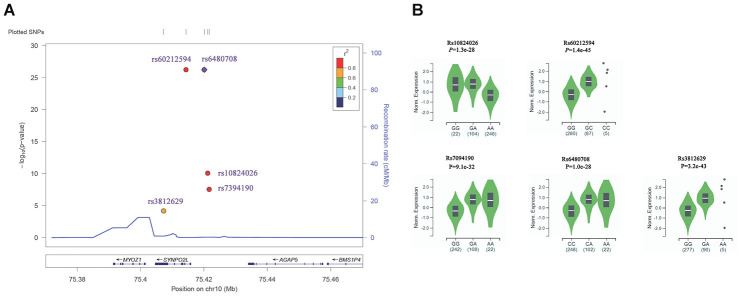
**Association of SNPs in 10q22 with AF and eQTL analysis.** (**A**) Regional plots for significant association with AF in 10q22. The *P* value was obtained from GWAS catalog database. SNPs plotted by their positions (UCSC hg19) on the corresponding chromosome against –log10 (*P*). Estimated recombination rates from 1000 genomes EUR populations were plotted in blue to reflect the local linkage disequilibrium (LD) structure on a secondary *y* axis. The most significant lead SNP (diamond) is denoted with the SNP identification number. Flanking SNPs (circles) are color-coded to represent the pairwise *r*^2^ measure of LD with the lead SNP: red, *r*^2^ ≥ 0.8; orange, 0.6 ≤ *r*^2^< 0.8; green, 0.4 ≤ *r*^2^<0.6; light blue, 0.2 ≤ *r*^2^<0.4; blue, r2< 0.2. These plots were generated by Locuszoom (https://statgen.sph.umich.edu/locuszoom/). (**B**) eQTL analysis showed that the association between AF susceptibility SNPs in 10q22 with the expression level of *MYOZ1* in human atrial appendage tissues (n=372). eQTL analysis were performed using GTEx data.

Another interesting finding is about AF susceptibility loci in 10q22, which was reported as the first genetic locus for familial atrial fibrillation by Brugada R et al in 1997, and SNPs including rs10824026 [28, 44], rs7394190 [21], rs6480708 [17] and rs60212594 [17] in 10q22 and upstream of *SYNPO2L* gene were found robustly associated with AF in several GWAS project. What is more, a missense variant in *SYNPO2L*, rs3812629 (p.Pro707Leu) was found to confer risk to AF in the Framingham population by Whole Exome Sequencing in Atrial Fibrillation [25] (Figure 6A). However, our eQTL analysis using GTEx data showed that all these GWAS positive AF SNPs including rs10824026, rs7394190, rs6480708, and rs60212594 were strong associated with the expression level of *MYOZ1* in human atrial appendage tissue with a *P* value from 1.3x10^-28^ to 1.4x10^-45^ (Figure 6B). Furthermore, the missense variant in *SYNPO2L*, rs3812629 (p.Pro707Leu), which confer risk to AF, was also found associated with *MYOZ1* expression level in human atrial appendage tissue, and the median normalized expression level of *MYOZ1* in homozygous risk allele GG carriers was -0.28 and extremely lower than in heterozygous GA carriers (0.94) (Figure 6B). *MYOZ1* encode myozenin 1, which is an intracellular binding protein involved in linking Z-disk proteins, and was known as a calcineurin-interacting protein, and help tether calcineurin to the sarcomere of skeletal and cardiac muscle [74–76]. Mutations in *MYOZ1* were found in the patient with dilated cardiomyopathy [77–78]. These results suggested that *MYOZ1*, but not *SYNPO2L* is the causal gene of AF.

Previously genetic studies in familial or sporadic AF have identified numerous mutations or rare variants that putatively cause AF [5, 79–83], and to recently, 44 genes that putatively cause AF were mapped to pathway of ion channels/ion channels related (*ABCC9, HCN4, KCNA5, KCNE1, KCND3, JPH2, KCNE2, KCNE3, KCNE4, KCNE5, KCNH2, KCNJ2, KCNJ5, KCNJ8, KCNK3,KCNN3, KCNQ1, RYR2, SCN1B, SCN2B, SCN3B, SCN4B, SCN5A, SCN10A*), transcription factors (*GATA4, GATA5, GATA6, NKX2-5, NKX2-6, PITX2, SHOX2, SOX5, TBX5, ZFHX3*), myocardial structural components (*GJA1, GJA5, LMNA, MYH6, MYL4, SYNE2*), signaling, protein turnover and others (*GREM2, NPPA,SH3PXD2A, PLN*). Compared to the list AF susceptibility genes including the 328 eQTL target genes what we have identified and combined with the closest gene of GWAS index SNPs, only 10 genes including *HCN4, KCND3, KCNJ5, KCNN3, PITX2, TBX5, ZFHX3, GJA1, SYNE2, SH3PXD2A*, and *PLN* were found have bath rare variants and common variants related with AF. These may result from most mutation screening were carried out in familial AF, early-onset AF or lone AF, and AF patients in GWAS were more complex.

In conclusion, we summarized the current results of the genetic studies including common variants identified by GWAS (338 index SNPs) and candidate gene based association studies (40 SNPs), and performed a comprehensive annotation of all these AF susceptibility loci found by GWAS and candidate gene based association. We identified 4 AF susceptibility SNPs in UTRs may change the microRNA binding ability, and 250 AF susceptibility SNPs in non-coding regions were identified as located in enhancer regions or might affect the histone mark of promoters and enhancers, 65 SNPs may change the situation of interaction with transcription factors and totally 40 transcription factors were found interact with these SNPs. Our results also showed that 151 SNPs can affect the expression levels of a total of 328 target genes and 81 of them associated with the expression levels of the closest gene. Long-range transcriptional regulatory function predictions showed that 211 SNPs interact with distal target genes, and 104 of them exert as an eQTL effect. We also performed a GO and pathway enrichment of the AF eQTL genes. Taken together, our study suggested the relationship between genetic variants and possible targeted genes, and provides insight into potential genetic pathways underlying AF incidence and development.

## MATERIALS AND METHODS

### Acquisition of AF susceptibility variants and search strategy

The workflow of the current study is presented in Figure 1. First, results of the current GWAS of AF were extracted from three public databases, including GWAS catalog (https://www.ebi.ac.uk/gwas/), GWAS central (https://www.gwascentral.org) and phenotype–genotype integrator (https://www.ncbi.nlm.nih.gov/gap/phegeni). We also searched the literature in Pumbed (https://pubmed.ncbi.nlm.nih.gov) to include all studies of AF GWAS. The keywords include atrial fibrillation, genome wide association or GWAS.

Besides the GWAS, several candidate gene based association studies have also identified a panel of genetic variants confer risk to AF. Results of these associated genetic variants were obtained by searching from the PubMed, EMBASE (https://www.embase.com) and Medline (https://www.nlm.nih.gov/bsd/medline.html) (Figure 1), and the searching keywords of medical subject headings (MeSH) including “atrial fibrillation” combined with “polymorphism, polymorphisms, variant, variants, single nucleotide polymorphism, single nucleotide polymorphisms, SNP, SNPs”. The results of literature searching were eligibility screened by two reviewers based on titles and abstracts. Studies published between 1 January 2007 and 1 November 2019 were included. Only case control association studies or cohort-based prospective studies were included. Functional researches, animal model studies or studies not performed in a population were excluded.

Information of AF GWAS index SNPs was extracted from the database of GWAS catalog, and the threshold of significant level for the association was set as *P* value lower than 5×10^-8^. For the SNPs from candidate gene based association studies, publications were reviewed by two reviewers independently and extracted the information about the variant(s) and the details of the population. Discrepancies in data extraction were resolved by discussion or submitted to a third reviewer if required. We divided the variants analyzed in candidate gene based studies into three groups, (i) replication study of the GWAS identified susceptibility loci of AF, (ii) novel variants with minor allele frequency (MAF) ≥0.1% (according to 1000 genome phase III global data), (iii) rare variants with a low frequency (MAF<0.1%) associated with AF by candidate gene association study or mutation screening. In our study, we excluded rare variants and mutations in (iii) from our further annotations, for the causal genes harbored mutations or rare variants of AF which were found in families or cohort were well summarized in previously reviews [8, 84] The significant level for SNPs in candidate gene based association studies was set as satisfying the Bonferroni correction. To reduce the probability of false positives, we exclude case controls studies if the statistical power <70%. The power was extracted from publications or calculated by PS: Power and Simple Size Calculation software [85].

### Genomic region annotations

All AF susceptibility SNPs including index SNPs identified by GWAS and SNPs identified by candidate gene based association studies were first annotated using Variant Effect Predictor (http://asia.ensembl.org/Homo_sapiens/Tools/VEP, GRCh38) in Ensembl to obtain their genomic region information.

### Functional annotation of AF susceptibility SNPs in exon

According to the genomic region information obtained from Variant Effect Predictor, non-synonymous SNPs were directly recognized as functional variants. SNPs in untranslated region (UTR) were analyzed the microRNA binding ability using MirSNP tool (http://bioinfo.bjmu.edu.cn/mirsnp/search) [86] and miRNASNP v2.0 (http://bioinfo.life.hust.edu.cn/miRNASNP2/) [87].

### Enhancer prediction and transcription factor (TF) binding sites prediction of AF susceptibility SNPs in non-coding regions

Splicing variants identified by Variant Effect Predictor were classified as functional SNPs directly. Next, for intronic or intergenic SNPs, the chromatin states data from the Roadmap and ENCODE to analyze whether they are overlapping any enhancers in possible AF related tissues and cell types.

For the AF susceptibility SNPs in the non-coding genomic regions, including in UTR, promoter, intron, and intergenic regions, SNP2TFBS database (http://ccg.vital-it.ch/snp2tfbs/) was used to predict potential binding ability between SNPs and transcription factors [88].

### Histone modification analysis

SNPs in promoter, intron, and intergenic regions may modify the histone binding ability, and here, using HaploReg (version 4.1) (https://pubs.broadinstitute.org/mammals/haploreg/haploreg.php) [89], we analyzed whether the identified non-coding AF associated SNPs overlap the major histone modifications (H3K9ac and H3K4me3 for promoter regions, H3K27ac and H3K4me1for enhancer regions) in AF related tissues and cell types.

### Long-range transcriptional regulatory function predictions

SNPs in the noncoding region may reside within or near regulatory elements controlling the expression of distal target genes through topological interactions, and using 3DSNP [90], we annotated the possible regulatory effect of identified AF associated SNPs by examining their 3D interactions with distal genes mediated by chromatin loops.

### Expression quantitative trait loci analyses

Genotype-Tissue Expression (GTEx) data were used in determining whether identified AF associated SNPs affect gene expression levels. eQTL analysis were performed bases on raw RNA-Seq data (RPKM) by genes from the GTEx V6 analysis freeze (dbGaP Accession phs000424.v6.p1) and included 25 tissues.

### Gene ontology (GO) and pathway enrichment analysis of eQTL targeted genes

Gene Ontology including biological process, molecular function, and protein class were annotated using PANTHER (http://www.pantherdb.org). KEGG pathway enrichment analysis were used in the Search Tool for the Retrieval of Interacting Genes (STRING, v11.0, http://string-db.org).

## Supplementary Material

Supplementary Table 1
